# Supplementary figures and images for: Genomics of an endemic cystic fibrosis Burkholderia multivorans strain reveals low within-patient evolution but high between-patient diversity
Source: PLoS Pathog. 2021 Mar 15;17(3):e1009418. doi: 10.1371/journal.ppat.1009418 (PMC7993779; doi:10.1371/journal.ppat.1009418)

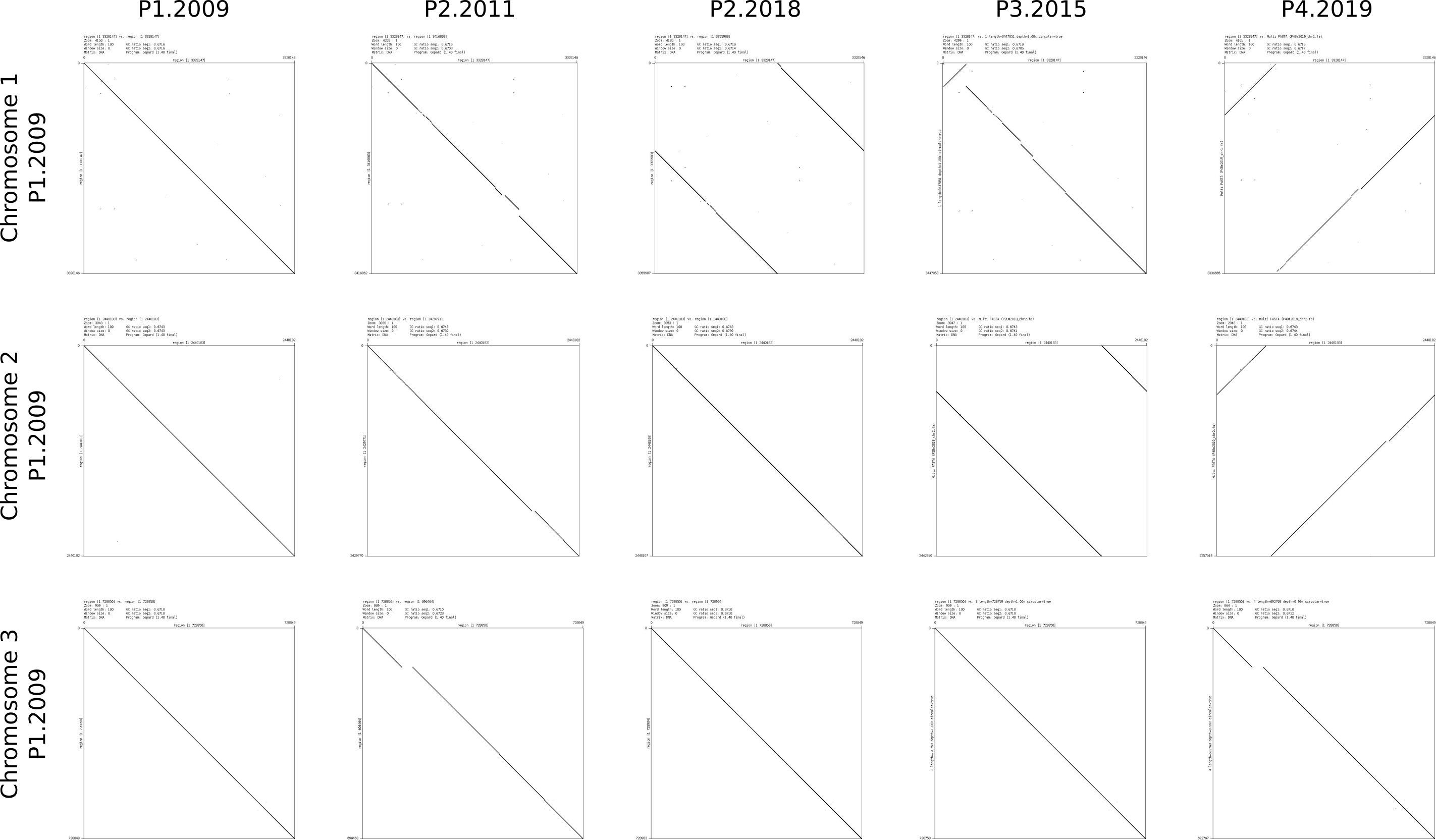

Supplement: S2 Fig — Chromosomes 1, 2, and 3 were compared in pairs using dot plots, highlighting the collinearity and overall sequence identity of the genomes across all isolates. Note that some chromosomes were not fully closed and subsequently rotated during the assembly resulting in visual artefacts including large inversions or re-arrangements. (PNG) [file ppat.1009418.s002.png]

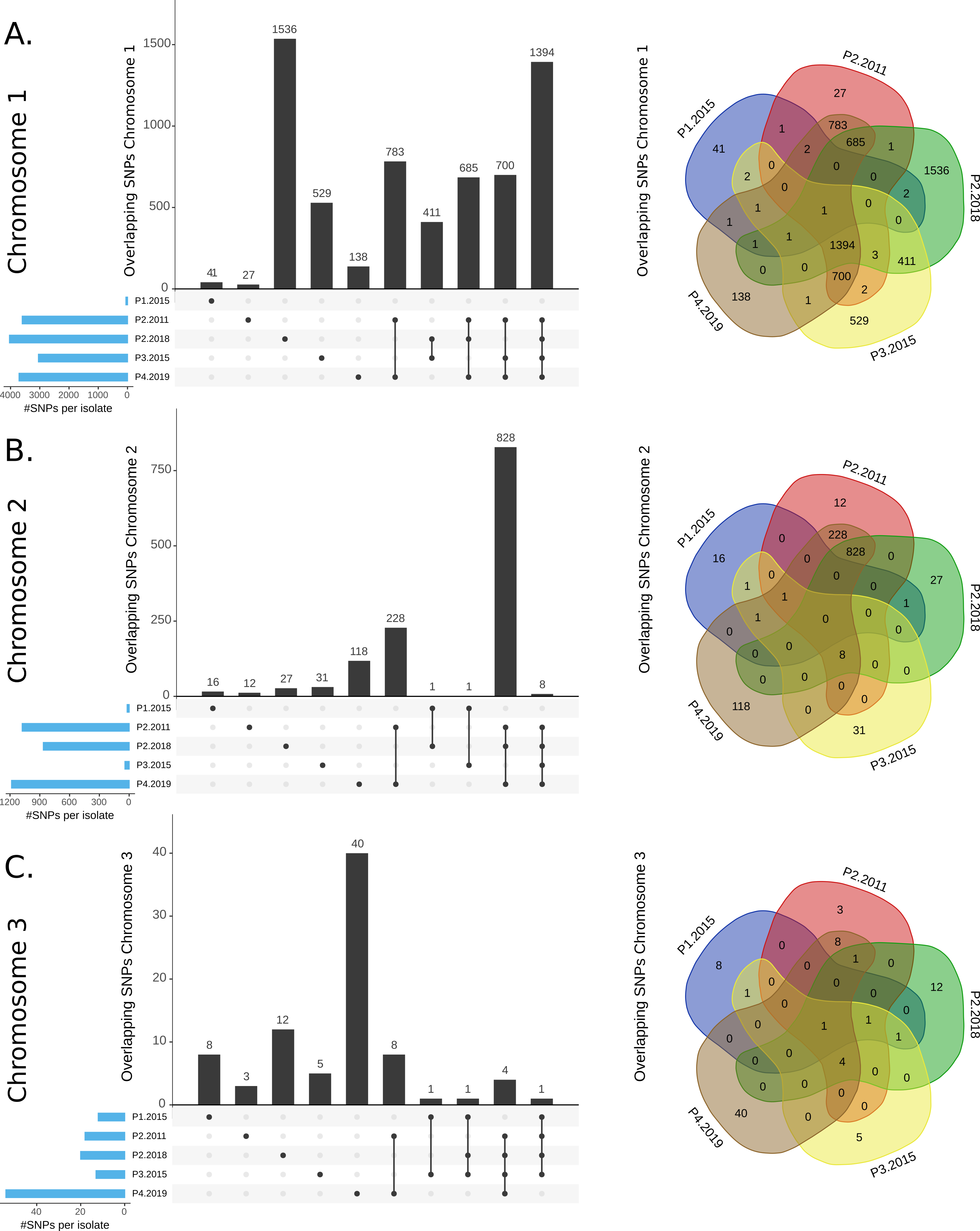

Supplement: S3 Fig — This visualization supplements Figs 5, 6 and 7 by delineating the sharing of SNPs found in the different isolates. For each chromosome, we provide two visualizations of the data using UpSetR and a standard Venn diagram. We collapsed the 85 SNPs found in the isolates from P1Bm2009 to P1Bm2015 and kept separate the P2Bm2011a,b and P2Bm2018 isolates from patient 2, as they differed by 1,990 unique (not shared) SNPs. (PNG) [file ppat.1009418.s003.png]

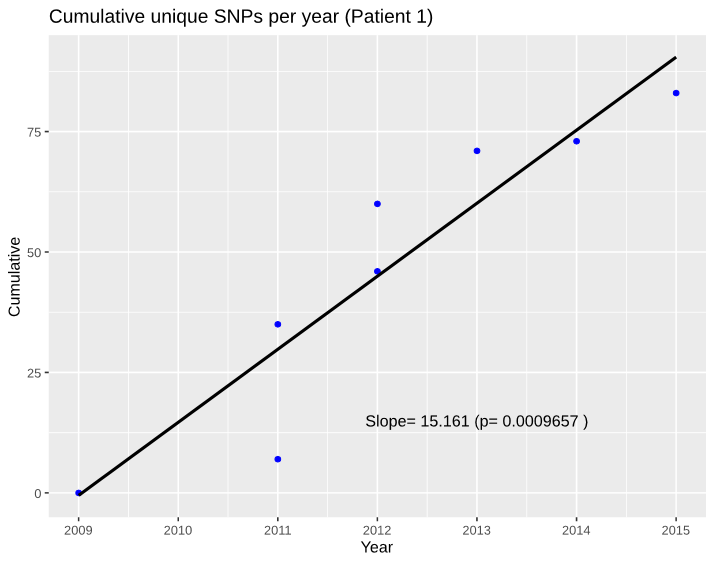

Supplement: S4 Fig — The cumulative SNPs are plotted over the time period of infection in patient 1. A linear model was used to estimate the rate of appearance of SNPs (15 SNPs/year). (TIF) [file ppat.1009418.s004.tif]
