# Supplementary material for: Genomics of an endemic cystic fibrosis Burkholderia multivorans strain reveals low within-patient evolution but high between-patient diversity
Source: PLoS Pathog. 2021 Mar 15;17(3):e1009418. doi: 10.1371/journal.ppat.1009418 (PMC7993779; doi:10.1371/journal.ppat.1009418)
Supplement: Attachment — Submitted filename: ResponseToTheReviewers_latest.docx [file ppat.1009418.s006.docx]

Cédric Lood, Ir.

Faculty of Bioscience Engineering

Department of Biosystems

KU Leuven

**RE: PPATHOGENS-D-20-02557, submission of revision**

**Please see below our response (italicized) to the reviewers comments (in bold) of the latest revision (PPATHOGENS-D-20-02557) :**

**Reviewer:	1**

**Part I – Summary**

**The manuscript by Lood *et al.* describes a genomic analysis of *Burkholderia multivorans* isolates recovered from persons with cystic fibrosis (CF). The authors conclude that the findings demonstrate limited genomic variation among isolates serially recovered from the same person, but high diversity among isolates recovered from different persons.**

**In general, this is a well written, nicely presented manuscript that clearly describes comprehensive genomic analyses of an important opportunistic human pathogen. The work is of interest on a few levels: (i) the analysis of serial isolates from two individuals furthers our understanding of bacterial adaptation/diversification during chronic human infection; (ii) the study nicely illustrates the strength of integrated long- and sort-read WGS approaches to assess genomic differences between related bacteria; and (iii) the work provides convincing evidence of independent acquisition of the same strain of *B multivorans* by multiple persons with CF, which adds to our understanding of the epidemiology of *Burkholderia* and is an important consideration in infection control in CF. The analyses, including antibiotic susceptibility phenotype, phylogenetic context of this strain, and detailed SNP analyses support the authors conclusions.**

***We thank the reviewer for her/his positive assessment of our manuscript.***

**Part II – Major issues**

**I have no major concerns with any of the analyses and believe the conclusions are well-justified.**

*Thank you.*

**Part III – Minor issues**

**1. Lines 39 and 291: SNP is typically used to describe single nucleotide polymorphisms (not small nucleotide…)**

***“Small” was indeed used instead of “single” because we also wanted to include indels (insertion/deletions). However, we concur this may be confusing and have therefore corrected this (L171-172 and L304 of the manuscript with tracked changes). The abbreviation of “SNP” has been removed from the abstract (L39-40 of the manuscript with tracked changes).***

**2. Line 53: necrosis, not necrotisis**

***This was corrected (L55 of the manuscript with tracked changes). The same issue was raised in question 2 of reviewer #2.***

**3. Line 70: “…higher RATES OF morbidity and mortality…”**

***The wording was changed as suggested (L73 of the manuscript with tracked changes).***

**4. Line 105: omit “in”**

***This was corrected (L108 of the manuscript with tracked changes).***

**5. Line 168: define ‘indels’ with first use**

***The full denomination « insertions or deletions » was added (L171 of the manuscript with tracked changes).***

**6. Line 338: elements, not element**

***This was corrected (L367 of the manuscript with tracked changes).***

**7. Line 348: omit “different”**

***This was corrected (L376 of the manuscript with tracked changes).***

**8. Methods: check spelling of antibiotics throughout: be consistent, use English spellings**

***The spelling of antibiotics was checked with the EUCAST spelling and corrected accordingly (Figure 2 and Material & Methods section “Bacterial isolates and patient data” of the manuscript with tracked changes).***

**9. Line 385: can a reference be provided for EUCAST PK/PD breakpoints?**

***A reference was added for the EUCAST PK/PD breakpoints (L414 of the manuscript with tracked changes).***

**10. Line 697: each originated from, instead of “were originating from”**

***This was corrected (L726 of the manuscript with tracked changes).***

**11. Figures 3, 5-7: can these be uploaded in higher quality; very difficult to read even with magnification**

***The figures that are attached to the reviewer’s pdf file are indeed compressed. However, the high-quality versions are available to the reviewers via the links on the top, right-hand corners of the page where the (previous) figures are located:***

***Figure 3 :***

[*https://www.editorialmanager.com/ppathogens/download.aspx?id=1533025&guid=79f8fea4-d6fa-4fab-b68b-8f21815d6ccc&scheme=1*](https://www.editorialmanager.com/ppathogens/download.aspx?id=1533025&guid=79f8fea4-d6fa-4fab-b68b-8f21815d6ccc&scheme=1)

***Figure 5 :***

[*https://www.editorialmanager.com/ppathogens/download.aspx?id=1533027&guid=33b7fed8-67fe-472e-859a-ea017474a5c4&scheme=1*](https://www.editorialmanager.com/ppathogens/download.aspx?id=1533027&guid=33b7fed8-67fe-472e-859a-ea017474a5c4&scheme=1)

***Figure 6 :***

[*https://www.editorialmanager.com/ppathogens/download.aspx?id=1533028&guid=b06494ea-12db-433f-8994-45d71f10761a&scheme=1*](https://www.editorialmanager.com/ppathogens/download.aspx?id=1533028&guid=b06494ea-12db-433f-8994-45d71f10761a&scheme=1)

***Figure 7 :***

[*https://www.editorialmanager.com/ppathogens/download.aspx?id=1533029&guid=5e24a2f2-d63b-4bf6-b34a-71be024f9aed&scheme=1*](https://www.editorialmanager.com/ppathogens/download.aspx?id=1533029&guid=5e24a2f2-d63b-4bf6-b34a-71be024f9aed&scheme=1)

**Reviewer:	2**

**Part I – Summary**

**Strengths
The manuscript "Genomics of an endemic cystic fibrosis *Burkholderia multivorans* strain reveals low within-patient evolution but high between-patient diversity" by Lood, Peeters, *et al.,* is well-written, the data well-analyzed, and the conclusions provided are sound.**

**The combination of two different sequencing technologies is important, giving a broader picture of what is happening genetically, than either technique can produce alone.

It is of concern that the number of SNPs detected per year are significantly higher than that previously reported for *Burkholderia cepacia* complex strains. This suggests that even without being hypermutator strains, the strains analysed were capable of much higher rates of mutation than previously known.**

**The report highlights the importance of tracking the genetics of sequential patient isolates. Significantly, although the antibiotic resistances from strain to strain did not vary appreciably, that is not to say that there were not mutations occurring and being selected for. For each infecting strain, perhaps it is only a matter of time before the correct combination of random mutations coalesce to produce a better adapted variant, whether that be SNPs, plasmid loss, prophage acquisition, or some other genetic event.**

**Weaknesses
Although, as with any genomics study aimed at inferring past adaptations, this retrospective study cannot truly know that the mutations observed were "adaptive". Given that similar recent studies showed an accumulation of almost completely different SNPs, it suggests not only as the authors' state ... different strategies for adaptation to the human lung ..., but also possibly that the random mutations (or that all the mutations) are not significantly adaptive.**

***We thank the reviewer for the positive assessment of our manuscript and acknowledge that not all mutations may be truly « adaptive ». However, following the suggestions of reviewer 3, we identified a set of 30 loci with mutations that appear in the isolates of at least three out of the four patients (see Table 4) and that enable us to hypothesize that the presence of these mutations across different patients highlights specific adaptations of the infecting strain.***

**Part II – Major issues**

**None**

*Thank you.*

**Part III – Minor issues**

**1) Line 50: In the author summary, the authors (surprisingly) indicate that " *multivorans* is the most prevalent species within the *Burkholderia cepacia* complex (Bcc) found infecting the lungs of patients with cystic fibrosis (CF)." Later, in lines 96 and 244, they go on to clarify that this fact is true for Belgium. In fact, although changes have occurred in some clinics and some countries recently, there are clinics and countries where *B. cenocepacia* is still the most prevalent Bcc species. Therefore, the authors should change the wording on line 50 to say "In some countires, *B. multivorans* is the most prevalent species ....".**

***Our apologies. We agree with the reviewer that this statement in the Author summary was incorrect and have amended this accordingly (L52 of the manuscript with tracked changes).***

**2) Line 53: the word "necrotisis is rarely used. The more highly preferred English word is "necrosis".**

***This was corrected (L55 of the manuscript with tracked changes). The same issue was raised in question 2 of reviewer #1.***

**3) Line 151: The authors refer to the "typical" conserved genome structure of Bm being three large replicons. If this structure is typical, then they are referring to previously determined data, and therefore should supply references for this work.**

***References to the seminal review by Mahenthiralingam et al. (2005) in Bcc and to the specific literature of B. multivorans (Peeters et al., 2017) were introduced (L155 of the manuscript with tracked changes).***

**4) Line 316: Or perhaps elsewhere. The authors indicate the locations of high SNP density in some of the replicons in some of the strains. The authors should comment on these stretches further. Why is this happening, What genes in common are being affected if any, and Why here and not everywhere. (Do these modified regions suggest improvements in adaptation, or merely tolerable unimportant mutations?)**

***This comment relates to question 5 of reviewer #3 and we agree the*** *reviewer’s point needs clarification.*

*As stated in* ***L319-334 (L339-344 of the manuscript with tracked changes)****, we could not link this to a hypermutator mechanism or genomic islands, and therefore suggest these high-density SNP regions are the result from recombination events.* ***A statement was added to elaborate in this (L353-358 of the manuscript with tracked changes).***

*Furthermore, we verified the affected gene content and added a statement to the results (****L229-232 of the manuscript with tracked changes, and Supplementary Table ST13****) to indicate that we did not find any particular interpretation of those results, which may indicate that recombination in these regions are tolerated by the strain (L345-L348 of the manuscript with tracked changes).*

**5) Line 416: It is apparent that PHAST can identify additional prophage element components not detected by PHASTER. Did the authors attempt to use this, or any of the other recent prophage software packages (e.g. ProphET, Prophage Hunter, PhageWeb, Phage_Finder, PhiSpy, LysoPhD, ) to analyze their data?**

***We previously developed a custom ensemble prophage annotation pipeline in our lab specifically for Pseudomonas aeruginosa prophages. We integrated different tools including PHASTER, prophET, Phigaro, and PhiSpy but our experience has been in general that PHASTER retains a good compromise in terms of sensitivity and specificity. Since, to our knowledge, PHAST lacks an automated query interface it was not considered in that pipeline. As such, we appreciate the suggestion of the reviewer and ran the genome through PHAST. All PHAST results were identical to the predictions from PHASTER, except for one additional incomplete prophage element which has been added to Figure 6 and Supplementary Table ST3.***

**Reviewer:	3**

**Part I – Summary**

**This is an exceptional comparative genomic analysis of a cluster of *B. multivorans* isolates belonging to a common lineage endemic to Belgium and capable of establishing chronic infections in persons with CF. The genomic methods are first-rate, attentive to structural variation and mobile elements, and show that this lineage is distinct from various others reported in this small field by undergoing patterns of adaptive evolution in vivo with limited overlap with prior studies. Another advantage is that four independent infections from this lineage are studied, enabling assessment of convergent evolution. Overall, this is a valuable contribution to the microbial genomic literature of Bcc, which remains relatively scarce.**

**Yet, the primary conclusion appears to be, as the abstract states, that these infections accumulate “peculiar patterns of genomic diversity between patients.” This leaves me with more questions than answers from this largely descriptive study, outlined below.**

***We thank the reviewer for her/his positive assessment of our work and for raising these interesting questions.***

**Part II – Major issues**

**1. If they derived from a common, unknown environment, do the changes in vivo indicate shared adaptations that distinguish infected isolates from the natural source, in effect enabling “triangulation” of the source environment?**

***This is an interesting idea, but unfortunately, we do not believe our dataset can be explored in this manner, since it assumes prior knowledge on the kind of mutations that are typical for a specific environmental niche. This has, to our knowledge, never been explored.***

**2. Is the natural environment a source for certain mobile elements that might be adaptive or maladaptive?**

***Thank you for this suggestion.***

***As stated in L181-199 of the manuscript with tracked changes, two plasmid groups were identified, suggesting this microdiverse B. multivorans ST-742 population appears to comprise different plasmids which can be dispensed in a human host. However, this disappearance could be interpreted as a sign of maladaptation or adaptation, i.e. whether the plasmid was previously useful in the natural setting or has become a burden during the colonization of the human lungs could not be derived from our data. No antibiotic resistance genes were detected on either plasmid and their loss may improve the fitness of the pathogen in vivo.***

**3. What are the phenotypic effects of any parallel genetic adaptations?**

***Excellent question! Thank you for bringing this up to our attention. Based on the reviewer’s idea, we performed a further analysis and identified 30 loci that had undergone mutations in at least three out of four patients (see new Table 4, L226-229 (results) and L337-339 (discussion) of the manuscript with tracked changes). Interestingly, only one of those loci was previously reported by Silva et al. (2016), further highlighting the different strategies for adaptation that can be used by different strains. However, this also reveals that some consistency in terms of adaptations to the CF lung can be found between isolates with a similar genomic background infecting different patients.***

**How exactly (genetically or phenotypically) is this novel ST different from other infectious isolates in CF or environmental isolates from your prior work?**

1) ***This is the first report of a microdiverse endemic strain in cystic fibrosis patients, spanning a 10-year time period, which makes our data rather unique by allowing us to glimpse, as suggested by the reviewer, parallel adaptations of a given strain in multiple patients.***
2) ***To our knowledge, strain ST-742 has not been isolated from the environment, and the true environmental reservoir of B. multivorans has not yet been elucidated (Peeters et al., 2016)***

***These points have been highlighted in the manuscript with tracked changes L119-121, L246-249, L317-321 (first point) and L159-169 (second point).***

**4. What can we learn about these dynamics about the epidemiology and evolution of opportunistic bacterial pathogens more generally? The manuscript offers little discussion outside our field of Bcc clinical microbiology, a missed opportunity.**

***We have deliberately opted to refrain from delving deeply into Bcc epidemiology. While interesting, we considered it too speculative and out of scope, based on the dataset generated.***

***To our knowledge this is the first study reporting an endemic strain in Belgium, and in the context of pathogen evolution, the first study dissecting the genomics changes of a given Bcc strain that has infected multiple patients. Our dataset is focused on four patients, but importantly we observe that the route of adaptation of the pathogen within patient is influenced by the genomic background of the strain. A hypothesis that builds on the work of Hassan et al. (2020) who discussed that different strains appeared to have different routes of adaptation within the patient.***

***We do hope and anticipate that the present study will encourage complementary studies on other B. multivorans lineages, other Bcc species and even other opportunistic pathogens (perhaps via collaborations with National Reference Centers). This in turn would enable the presented results to contribute to the broader field of epidemiology and evolution of opportunistic bacterial pathogens in general.***

**5. Given that a major discovery is of clusters of mutations in certain regions, which as the authors acknowledge suggest recombination events, what is the novelty? That recombination may happen perhaps due to coinfection in the airway or possibly in the reservoir prior to reinfection?**

***This comment also relates to question 4 of reviewer #2. As stated in (L222-226 of the manuscript with tracked changes), we observed different start positions of the otherwise similar, high-density SNP regions in isolates from different patients, and therefore hypothesize that these recombination events took place in the environmental reservoir, prior to infection and that they contribute to the microdiversity of the environmental B. multivorans ST-742 population. A statement was added to elaborate on this (L353-358 of the manuscript with tracked changes).***

**Part III – Minor issues**

**No comments from reviewer 3.**
